# Our Bureau of Information

**Published:** 1911-12-16

**Authors:** 


### OUR BUREAU OF INFORMATION.

### A Brotherhood of Personal Service.

We announced a week or two ago that we intended to form a Brotherhood of Personal Service. Everywhere around us we see hands held out for help, and other hands that would fain help them, but between the two intervenes a thick curtain—a mist through which each seeks the other but cannot find it. We want to make the two hands meet. It is a matter of organisation; for charity, like every other work, needs to be organised. We are quite aware that there is a general notion that organised charity means organisation with the charity left out; but this is not the case. It only means finding out and putting together the two halves of a beautiful whole. To illustrate what we mean, one can imagine a lady of moderate means, with a pleasant home, who is willing to help another less fortunate by giving her a place in her home at a cost that does not more than cover expenses. (There are many such.) And there are many who would be grateful for the offer. But if the first lady mentions her desire to any of her friends, she may be offered as an inmate of her home someone who can pay nothing, or a mental patient, or a person whose antecedents, though good and honourable in themselves, are not such as to make her a companion for a gentlewoman. And meanwhile there are scores—perhaps hundreds—to whom such an offer as this would be the greatest of boons. Can we put the two classes in touch with each other? We believe it is possible, and it is in this that we ask our readers to help.

Again, we are asked our opinion about such-and-such a place—a place often remote from London. Now, it is obviously impossible for the editor of a London newspaper to know about every nursing or private home in the kingdom, and impossible for him, except under rare conditions, to send a member of his staff to investigate. We are doing our best to make sure that no institution shall be placed on our list of approved homes that is not satisfactory, but it is obviously impossible to make a personal investigation of every place, from John o' Groats to Land's End, that offers to receive patients. We already make every possible use of local knowledge when we can get in touch with it, but there is at present an inevitable delay in making our inquiries. How different it would be if within a few miles of the place we had a member of the Brotherhood to whom we could refer for information! We ask from our members no expenditure of money, but we do ask from them the expenditure of time and energy and goodwill. But surely there are many people who are willing to give this. And, on the other hand, they will be able to get the best and fullest information in regard to the assistance of any case in which they are interested.

### Approved Homes.

It is in connection with this that we desire to form a list of Approved Homes. We have had, since we commenced this Bureau, a large number of communications from managers of homes. Now, it is impossible for us to take the word of any owner of a home without some reference. The home may be admirable—doubtless it often is—but we can have no assurance of the fact from the bare word of the person who is at the head of it. We therefore ask that those who wish to be put on our list shall send us at least two testimonials, and also that these should be of fairly recent date, and should refer to the home, and not merely to the previous career of the head of it. It is no new thing for this journal to insist on the differentiation of capacity. We have for many years pointed out that a good nurse may not be a good sister or matron; that one who is admirable in hospital work may not be successful in district or private nursing. Similarly a lady may be a splendid nurse without having in addition those housekeeping qualities which would make her a successful head of a home. Therefore we shall give special heed to testimonials from local doctors and from patients who have stayed in any home which seeks a place upon our list. We shall, in addition, try to get an independent local opinion, but we wish to point out to applicants that local and recent testimonials are of great importance in settling the claims of a home.

### What You Give and What You Get.

From those whom we are prepared to put on our List of Approved Homes we ask a registration fee of 5s. On payment of this sum the name of the home and of the head of it will be at once published in our Bureau, and the list will be repeated quarterly—i.e. it will be published in our issue of the first Saturday after each quarter-day. In addition, we propose to have the list, as revised and increased, printed annually, and it will be sent to inquirers when they apply to us for information. This will not preclude us from sending other answers to any application, but it will give a guarantee for the merits of the homes on the list. It may be necessary to state that though, no doubt, the majority of the homes on the list will be those which are founded on a remunerative basis, we shall be quite as careful in our inquiries about those which are set up on a charitable or semi-charitable footing—i.e. those which ask for a small payment, but one which cannot be regarded as absolutely profitable. There is a great demand—and one which includes many touching cases—for homes where people of refinement can be received at moderate rates—rates which only cover necessary expenses, or but little more. That is a class whose needs we specially desire to meet, but not at the expense of efficiency. We will recommend no place which we do not believe, after having made the best investigations we can, to be suited to the needs of those to whom we recommend it.

There is great need, and there is much assistance. Help us to bring the two together.

### Needs and Helpers.

"Perseverance" writes: I have for some time been collecting for sale articles of various sorts made by some really poor patients. I have some crochet-work, pillow lace, macramé lace, drawn-thread work, table-cloths and tea-cloths done by them. Could you find me some willing purchasers? Specimens sent on approval.

[The Brabazon Society, which encourages and sells the work done by patients in infirmaries, might be able to help you. There is an annual sale at your Union Infirmary, and you might be able to get your patients' work included in it. Consult some of the lady Guardians.]

"Aberdeen" writes: I am interested in an orphan boy, and would like to get him into a home where he would be well trained for after life; but I have only recently come here, and do not know much about Scottish institutions. Is there anything in Scotland corresponding to Dr. Barnardo's Homes? It would be better for him to be in Scotland, as his relations, though they cannot maintain him, would have him for holidays.

[The Orphan Homes of Scotland, at Bridge of Weir, Renfrewshire, would be suitable for this case. Superintendent, Mr. J. Quarrier.]

"Molly Mo" writes: For four years I have had a home for sick, backward, and slight mental cases (children), and have been very successful with even what seemed rather hopeless cases. But I have taken cases at a very low rate, and would like some that could pay larger, although still moderate fees.

[It is rather difficult to mix cases at different rates, as all must have practically the same treatment; but we have sent your name to one of our applicants, as your home seems to meet the requirements of the case.]

We regret that by mistake the Nightingale Nursing Home, Hanley Road, Hornsey, was given in our issue of November 25 as one which would take chronic cases for less than £1 1s. a week. We are glad to be informed of the terms accepted by the Home.

Will C. Cox kindly send testimonials? if possible, one from a local doctor and one from a patient's friends.

